# Statin-Induced Necrotizing Autoimmune Myopathy: An Uncommon yet Challenging Complication of Statin Use

**DOI:** 10.7759/cureus.71311

**Published:** 2024-10-12

**Authors:** Bárbara Paracana, Carolina Amado, Jéssica Krowicki, Sérgio Monteiro, Mariana Sousa

**Affiliations:** 1 Internal Medicine, Unidade Local de Saúde da Região de Aveiro, Aveiro, PRT

**Keywords:** anti-hmgcr antibodies, elevated creatine kinase, myopathy, statin-induced necrotizing autoimmune myopathy, statins

## Abstract

Statin-induced necrotizing autoimmune myopathy (SINAM) is a rare, disabling, and potentially life-threatening complication of statin use. Although not fully understood, a prevailing hypothesis proposes that statins induce molecular changes in 3-hydroxy-3-methylglutaryl-CoA reductase (HMGCR), leading to the formation of anti-HMGCR antibodies. The subsequent myofibre necrosis is expressed as progressive and persistent proximal muscle weakness and elevated creatine kinase (CK) levels. The authors describe the case of a 49-year-old Caucasian male with the diagnosis of SINAM after two years of statin therapy. He presented with progressive proximal muscle weakness, particularly at the shoulder and pelvic girdle, and significant elevated CK levels. Besides statin suspension, treatment with three immunosuppressants was necessary with clinical and analytical remission after two years.

## Introduction

The atherosclerotic vascular disease has long stood as the leading global cause of mortality. One pivotal advancement in combating this disease has been the introduction of inhibitors targeting the 3-hydroxy-3-methylglutaryl-CoA reductase (HMGCR) enzyme, commonly referred to as statins. Although considered safe, muscle-related side effects of statins are well recognized [1]. It's now assumed that in exceedingly rare cases, statin treatment can trigger the development of an autoimmune myopathy known as statin-induced necrotizing autoimmune myopathy (SINAM). Although not fully understood, the most widely accepted theory suggests that statins may induce the overexpression of HMGCR in genetically susceptible individuals, leading to increased production of antibodies against HMGCR and subsequently resulting in a necrotizing myopathy [2,3-5]. Patients with SINAM usually experience progressively worsening proximal muscle weakness and markedly elevated muscle enzymes. Importantly, symptoms may begin after years of exposure and persist after cessation of statin, which should warrant further investigation to establish this diagnosis. The authors present a case of SINAM related to statin use and unravel, among other aspects, the diagnosis, treatment, and prognosis of this disease.

This article was previously presented as a poster at the 27^th^ Portuguese National Congress of Internal Medicine on October 4, 2021.

## Case presentation

A 49-year-old Caucasian male with a medical history of type 2 diabetes, dyslipidemia, and ischemic cardiomyopathy treated with atorvastatin 40 mg daily, aspirin 100 mg daily, and metformin 1,000 mg twice daily for the past two years presented to the ER. He complained of a one-month history of progressive proximal muscle weakness, particularly at the shoulder and pelvic girdle, fatigue, and weight loss. He denied fever, myalgias, arthralgias, skin, nails, and hair alterations, recent travel, trauma and infections, diet alterations, or newly prescribed medications or vaccines.

There was no personal or familial history of autoimmune or neuromuscular diseases. During the physical examination, the patient displayed an abnormal range of motion in both upper and lower extremities, with 4/5 muscle strength and preserved sensation.

Laboratory assessments revealed elevated creatine kinase (CK), aspartate aminotransferase (AST), and alanine aminotransferase (ALT) levels (Table 1). Inflammatory markers, creatinine, ions, vitamins, bilirubin and alkaline phosphatase levels, and thyroid function were normal. Viral serology was negative. Full-body CT scan, echocardiogram, and endoscopies revealed no abnormalities. Pulmonary function tests demonstrated restricted function. Further study revealed elevated aldolase level and a positive anti-HMGCR antibody (Table 1). Electromyography findings demonstrated a symmetrical moderate-to-severe inflammatory myopathy primarily affecting the proximal domain of the upper limbs. A muscle biopsy was performed showing various stages of muscle fiber necrosis with myophagocytosis focus and with a discrete lymphocytic infiltrate present. A diagnosis of SINAM was assumed.

**Table 1:** Key initial and additional laboratory assessments NRV: normal range values

Laboratory assessments	Results	NRV
Creatinine kinase (CK)	9730 U/L	46-171 U/L
Aspartate aminotransferase (AST)	432 U/L	13-18 U/L
Alanine transaminase (ALT)	657 U/L	10-49 U/L
Aldolase	133 U/L	< 32.0 U/L
Anti-HMGCR antibodies	>200 UA	<20 UA

Despite statin suspension and aggressive fluid therapy, a sustained plateau in CK levels was observed. Prednisolone 60 mg/day was started (1 mg/kg/day). Due to limited clinical and analytical response, after two weeks, methotrexate was added at a weekly dosage of 15 mg, along with daily administration of 5 mg of folic acid. After two months, due to only partial improvement despite dual immunosuppressive therapy, rituximab was initiated. With triple therapy and physiotherapy, the patient progressively regained his functional capacity. Additionally, CK levels showed a decreasing trend in the subsequent months, but normal values were only achieved after one year.

**Figure 1:**
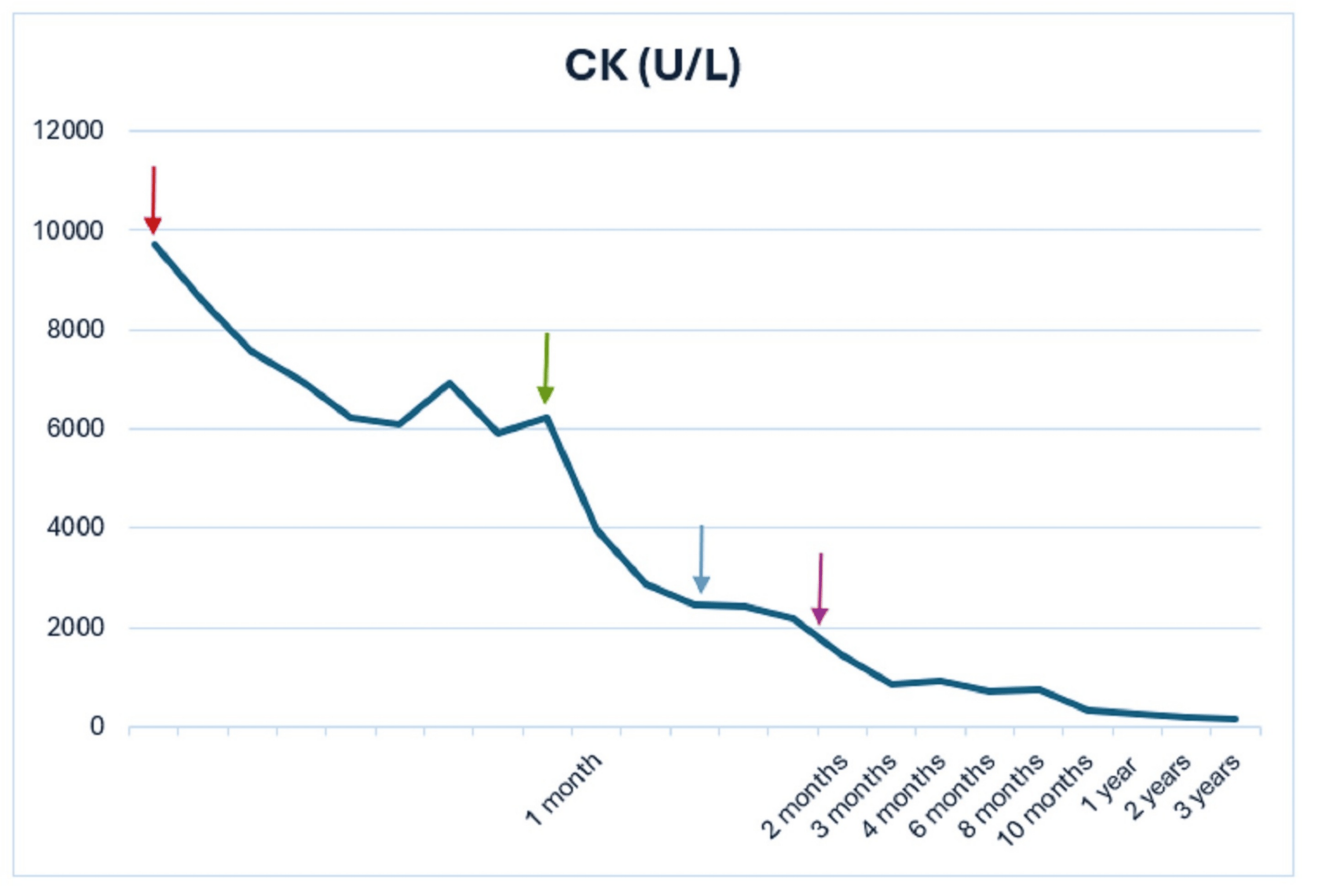
Graphical representation of the evolution of creatine kinase (CK) values during the clinical course Arrows indicate key interventions: red (statin discontinuation), green (corticosteroid initiation), blue (methotrexate initiation), and purple (rituximab initiation)

In summary, the patient received two doses of rituximab, administered in the second and third months post diagnosis, and corticosteroid therapy was gradually tapered and discontinued after one year of treatment. Lastly, methotrexate was maintained for two and a half years (Figure 1). Four years after the diagnosis of SINAM, the patient has completely recovered his muscle strength.

## Discussion

Statins inhibit HMGCR and are widely used to lower cardiovascular risk [1]. Approximately two to three in 100,000 patients treated with statins will have SINAM as an adverse event [5]. As in the presented case, SINAM is characterized by severe symmetric proximal muscle weakness and significantly elevated creatine kinase levels, which persist even after discontinuing statin therapy. Additionally, patients may experience fatigue, cramping, stiffness, and tendon pain [3-5]. Complications arising from muscular compromise can manifest in various ways, such as dysphagia, requiring nasogastric tube feeding, or dyspnea due to diaphragmatic muscle involvement, potentially leading to respiratory failure [2]. Extra-muscular manifestations are rare but may include fever, skin rash (such as Gottron’s sign), and interstitial lung disease [6].

The histological characteristics of SINAM in biopsy samples include necrotic muscle fibers distributed with a scattered pattern at various stages of necrosis, myophagocytosis, and regeneration. Cellular infiltration, primarily located in endomysia and perivascular areas, consists mainly of macrophages. Ultimately, a diagnosis is confirmed with positive anti-HMGCR antibodies. Though not fully elucidated, several theoretical models have been proposed to explain the underlying mechanism of HMGCR autoimmunity. One model suggests an association between the DRB1×11:01 allele and the development of anti-HMGCR autoantibodies. Statin exposure may enhance HMGCR expression in regenerating myofibers in genetically predisposed individuals. Evidence indicates the presence of HMGCR proteins on muscle fiber sarcolemma, leading to complement protein activation. Additionally, statin-HMGCR interaction may produce non-immune cryptic epitopes, perpetuating autoimmune responses even after statin cessation [2-4, 7]. Some factors listed as risks for the disease include being over 50 years old, of African American descent, having decompensated liver disease, severe renal disease, and uncontrolled diabetes mellitus [4]. Anti-HMGCR autoantibodies can also be detected in individuals with autoimmune myopathy who have never received statin therapy. They typically exhibit a younger age, myopathy, and are less responsive to treatment compared to statin-exposed patients [5,7].

Different cohorts have reported a mean interval of three years between the initiation of statin therapy and the onset of SINAM symptoms. However, this onset duration can vary, ranging from as brief as two months to as prolonged as 14 years. Furthermore, documented instances exist wherein SINAM emerges several months following the discontinuation of statin therapy. Some authors believe that specific statin exposure is a contributing factor, with atorvastatin being the most cited in published literature. However, cases have also been documented with rosuvastatin, simvastatin, fluvastatin, and pravastatin. Conflicting data exist regarding the correlation between serum anti-HMGCR titer and markers of disease activity or treatment response. Longitudinal studies have indicated that anti-HMGCR titers may decrease with treatment, but they rarely normalize, even in patients who regain normal muscle strength [8].

Treatment

Treatment approaches for SINAM are primarily based on case series, observational cohorts, and expert recommendations due to the lack of clinical trial evidence. There are some rare reports of improvement with statin discontinuation [6, 9], nevertheless, immunosuppressive therapies are the cornerstone of treatment, often requiring a combination of at least two or three agents to achieve disease remission [5, 9], as in the case reported. The consensus guidelines established during the 224^th^ European Neuromuscular Centre International Workshop in 2016 advise initiating glucocorticoid therapy as the first-line treatment, either orally or intravenously for severe cases. Methotrexate should be initiated concurrently or within a month, with azathioprine or mycophenolate mofetil as alternatives. Additionally, IVIg may be considered an adjunctive therapy. If there is an inadequate response within six months, the addition of rituximab is recommended. Once disease control is achieved, glucocorticoid doses should be tapered to the lowest effective dose, and methotrexate and/or rituximab should be continued for a minimum of two years [10]. Refractory patients can be trailed on immunosuppressants azathioprine, mycophenolate mofetil, and/or abatacept to help maintain remission [4]. Prognostically, younger patients tend to have a poorer outcomes, exhibiting more severe muscle weakness and higher CK levels [3] when compared to their older counterparts. Interestingly, older patients demonstrate a faster rate of strength improvement than younger ones, even after adjusting for potential bias [6]. Some patients who have undergone treatment regain full strength despite persistently elevated CK levels, suggesting an ongoing but mitigated process where muscle regeneration exceeds muscle destruction. However, there is ongoing debate regarding whether therapy should be intensified in such cases. In some patients, muscle weakness persists even after normalization of muscle enzyme levels, probably explained by the prolonged undertreatment, leading to permanent muscle damage and fatty replacement of muscle tissue [5].

## Conclusions

For most patients, statins are a safe and essential treatment for managing cardiovascular risk. However, although rare, serious musculoskeletal adverse effects such as SINAM can occur. Upon clinical suspicion, statin therapy should be promptly discontinued. Advances in the detection and quantification of anti-HMGCR antibodies have enhanced the accuracy and accessibility of diagnosis. Effective management of SINAM typically involves aggressive immunosuppressive regimens, as demonstrated in this case with the use of a combination of corticosteroids, methotrexate, and rituximab. When these treatments are initiated early, they can lead to a favorable prognosis, including significant recovery in muscle strength and functional capacity, as observed in our patient. Continuous long-term follow-up is essential for monitoring therapeutic response and making necessary adjustments. Early clinical suspicion and prompt intervention are critical for altering the disease course and optimizing patient outcomes.
